# Review: Improving global food security through accelerated plant breeding

**DOI:** 10.1016/j.plantsci.2019.110207

**Published:** 2019-10

**Authors:** Bert Lenaerts, Bertrand C.Y. Collard, Matty Demont

**Affiliations:** a UHasselt, Centre for Environmental Sciences, Hasselt, Belgium; b International Rice Research Institute (IRRI), Metro Manila, Philippines; c Yanco Agricultural Institute, NSW Department of Primary Industries, Yanco, Australia

**Keywords:** RGA, rapid generation advance, MAS, marker-assisted selection, DALY, disability-adjusted life year, Food security, Accelerated plant breeding, Rice, Rapid generation advance, Technology adoption

## Abstract

With an expected 9 billion people by 2050 and average income on the rise in the developing world, meeting future food demand will be a challenge. Climate change, urbanisation and land degradation are putting further pressure on the food supply. The multifaceted and self-reinforcing nature of these challenges calls for a fundamental transformation of the food system. In the past, crop improvement through breeding has been the major tool to lift people out of poverty and increase global food supply. To adequately address these food security challenges, new improved crop varieties need to be developed and reach farmers sooner as a partial solution. In this review, we focus on various proven conventional and biotechnological accelerating plant breeding methods that do not require genetic engineering or gene editing. We pay specific attention to the feasibility for implementation by national agricultural research systems in developing countries in the short term. We argue that postponing technologies that can accelerate breeding makes no economic sense and justify immediate adoption of accelerated breeding practices in the public sector. Considering a wide range of factors including the economics of accelerated breeding, we advocate the use of a method called rapid generation advance (RGA) as the most feasible method for accelerating breeding in the public sector.

## Highlights

• A shift in breeding mentality is needed to realise improved varieties’ potential to increase food security.
• Information on markets, environment and climate, pre-breeding research and effective dissemination methods are needed too.
• Rapid generation advance has the highest adoption potential of all the accelerated breeding methods in the public sector.
• Foregone benefits from earlier adoption could have mitigated the long-term negative impact of hunger on human development.
• Postponing accelerated breeding technologies makes no economic sense and immediate adoption is economically optimal.

## 1 Introduction

**Fig. 1:**
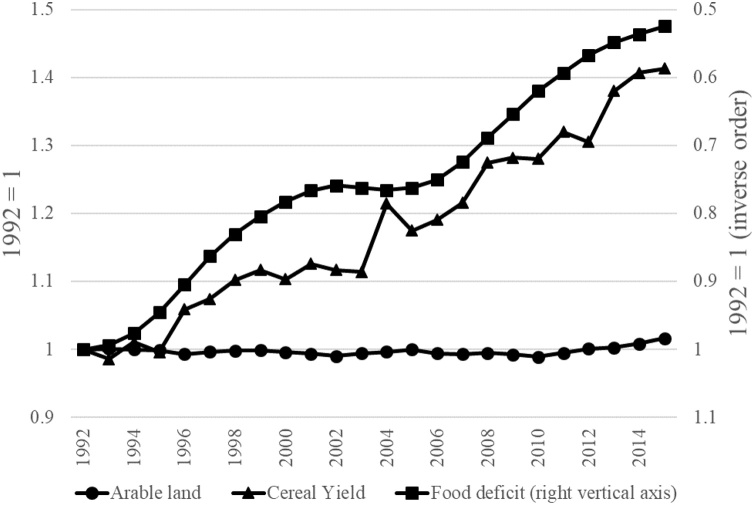
Time trend in arable land available, cereal yield and food deficit (kilocalories per person per day).

*Source*: World Bank Development Indicators 2019 [2].

Recent decades have seen an unprecedented rise in cereal production and a consequent drop in world hunger. The intensity of the food deficit has decreased by 50% since 1992 (Fig. 1) bringing the number of undernourished people at 820 million, still a substantial amount. Although impressive, this historic trend might give the false impression that improvement in food security is the current trend. In contrast, recent evidence has shown that the number of undernourished people has risen since 2014—both in absolute and in relative terms. The number of undernourished people in Africa, Western Asia and Oceania is larger now than it was a decade ago [1].

The most pressing challenges to food availability now and in the future are population growth and climate change. Population levels are rising fast [3] fuelling global demand and putting pressure on land due to urbanisation [4,5]. Access to food crucially depends on economic growth, which is still important in alleviating hunger (both chronic and hidden) [6] and poverty [7], although it might not be sufficient to accelerate reduction of hunger and malnutrition [8].

Climate change is widely considered to be a major threat to future food security. Although the exact consequences of climate change are impossible to predict, the general view is that global crop production will be negatively affected [[9], [10], [11], [12]]. Adverse effects may happen through increasing levels of CO_2_, temperature [11,[13], [14], [15]], pests and diseases [16], and deteriorating head and milling yield, and quality attributes [17]. Climate change also increases the frequency of extreme weather events such as droughts and floods [13,18,19]. Thus, future food security faces a four-fold challenge: upward pressure on demand, downward pressure on supply and the need for production that is both resilient and sustainable [20,21]. Furthermore, these factors do not simply add up; due to their interaction and collective reinforcement, they are expected to amplify the overall burden of food insecurity and consequent need for transformation of the food system [22].

While necessary increases in food availability seem daunting, they are not unprecedented. Since 1960, the total production quantities of rice and wheat have more than tripled [23]. Fig. 1 shows how the reduction in the food deficit was achieved through the progress in cereal productivity. In general, the annual rate of yield increase—attributed to improved varieties—was reported to be approximately 1% in rice [24], wheat [25], barley [26] and oats [27].

Future challenges to the production environment will make it difficult to maintain the same rate of productivity increase in the future. Yet, the current rate of yield increase may not even be sufficient to meet future demands for cereal crops [28]. Most studies estimating the annual production increase needed to keep pace with demand project a necessary increase of more than 1%, superior to the past annual growth trend in yields [[28], [29], [30], [31], [32], [33]].

With demand outweighing supply, the number of undernourished people may increase again, reversing the progress made in alleviating hunger globally. Moreover, even if on average supply matches demand, sudden changes in climate and environment might lead to reduced incentives for investment in food systems and reduction in food security. Challenges such as growing demand and climate change will determine to a great extent the future state of food security and might hold back or reverse progress toward a world without hunger [34,35].

In this light, recent studies have argued that current food production practices do not suffice and therefore a transformation of the food system is required. Possible approaches to enhance future food availability include (i) a dietary shift [36], (ii) food waste reduction [37], (iii) closing yield gaps through improved agronomy [[38], [39], [40]], (iv) an increase in arable land [41], and (v) improved productivity [30]. Improvements in productivity critically depend on both development of new technologies (e.g. new crop varieties, precision agriculture, etc.) and their dissemination. In many cases, especially in the developing world, dissemination and adoption of new technologies is severely hampered, limiting their potential to improve food security.

We have argued that current food production practices—including business-as-usual breeding scenarios—do not suffice to meet future food demand. In this paper, we advocate accelerated breeding as a realistic partial solution to face the challenges ahead. Undoubtedly, there will need to be a holistic solution involving multiple disciplines (breeding, agronomy, pathology, extension, seed production, post-harvest technology); however, breeding is the logical starting point. Therefore, we discuss various proven conventional and biotechnological breeding methods that do not require genetic engineering or gene editing. We pay specific attention to feasibility for implementation by national agricultural research and extension systems (NARES) in developing countries in the short term, which may have limited capacity to implement advanced technologies. We justify the use of accelerated breeding—specifically rapid generation advance (RGA)—as a solution to future food security challenges by reviewing the associated costs and benefits.

## 2 Accelerated breeding

### 2.1 Breeding fundamentals

Plant breeding is a time consuming process due to the biology of crop species. It generally takes at least 10 years to develop and release a new rice variety [42]. The breeding process generally consists of three stages: hybridisation, line fixation and field trials. Plant breeding is a large-scale logistical operation involving thousands to hundreds of thousands of plants in the initial line fixation stage, but numbers are greatly reduced to a small selected number of advanced breeding lines by the end of the breeding process. Selection takes place during the breeding process, such that approximately 99% of the original starting material in a breeding program is rejected and discarded. Most countries have an independent government-led system for evaluating the “best” advanced breeding lines compared with the current varieties, which usually requires two years of testing.

Regarding variety development, it is important to emphasise key points regarding the time to develop a new variety. One time-consuming component is the “line fixation” stage. This is due to biology (i.e. genetics) as breeding material is not genetically uniform or “stable” (i.e. plants are not homozygous) until at least 6 to 8 generations (i.e. self-pollination events). Homozygous lines are required for testing in advanced field trials. Furthermore, time is required to produce enough seed during the breeding process (i.e. for subsequent field trials) because seed of a new breeding line originates from only a single plant. It generally takes about 10 years to develop a new field crop variety, although there are differences between crop species and varietal testing requirements across countries [42].

**Fig. 2:**
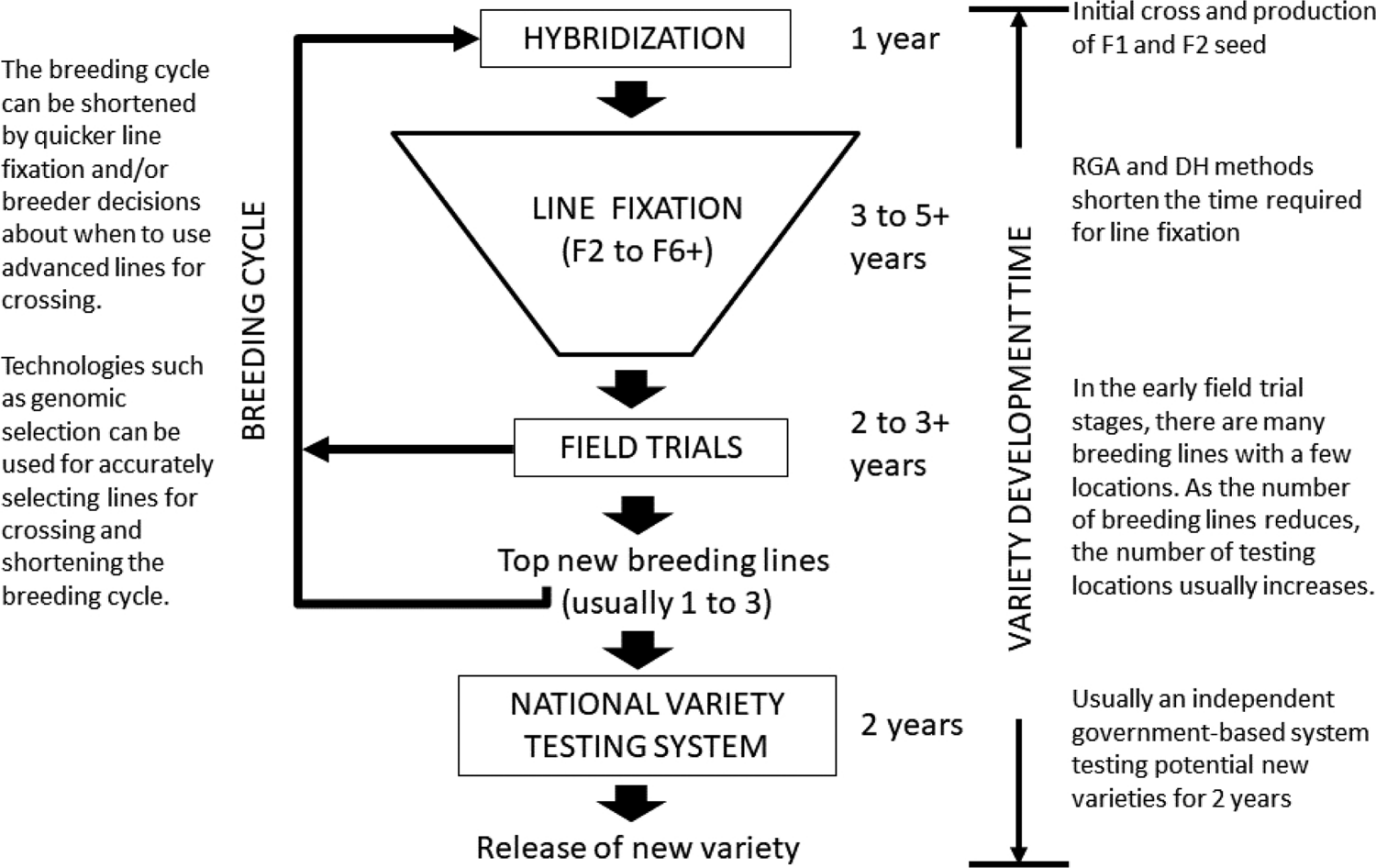
Simplifed overview of a typical breeding and variety release scheme for self-pollinated field crops. Most countries require independent testing for at least 2 years within a national testing system.

Another component of variety development is the breeding cycle. This refers to the time required for breeders to initiate development of a new variety, often referred to as the time “from cross to cross”. A summary of a typical breeding and variety release scheme for self-pollinated crops is shown in Fig. 2.

### 2.2 On breeding philosophy

To accelerate plant breeding, we argue that a new breeding philosophy is required. Fundamentally, plant breeders are applied scientists engaged in product development. Given the relatively long product development time for new varieties, we argue that breeders should attempt to accelerate breeding in order to generate products more quickly, and in order to be able to respond to unpredictable changes in crop production environments, climate or markets. The most obvious change plant breeders need to respond to is the emergence of new races and biotypes of pathogens and pests, that overcome resistance (usually major gene or “vertical” resistance) in current varieties [43,44]. Next, more consumer-focused approaches will be needed to make plant breeding more responsive to changing preferences and quality requirements in domestic and international markets [45]. In other words, breeders should implement ways to reduce the variety development time and breeding cycles. This is based on the assumption that the performance of the new varieties is not compromised by use of technologies to make breeding faster. It is worth noting that there are fundamental differences in breeding philosophy when comparing the public versus private sector, and considering breeding programs in developing versus developed countries. In our experience, private sector breeding programs are generally much faster than those in the public sector as private companies are motivated by “speed to market”, leading to faster profits.

Breeders have always used new methods and tools to develop new varieties, and accordingly, have long discussed the pros and cons of different breeding methods, especially with regard to the speed of breeding [46]. In recent decades, there have been many technological developments in a range of areas applicable to crop research: molecular genetics and genomics, genetic modification, trait physiology, phenomics, and geographical information systems [47,48]. In the next two sections, we reviewed literature with the specific intention to evaluate proven technologies for accelerated breeding that are suitable for implementation in breeding programs in the public sector in the short term. We delimit the scope of our review to technologies that do not require genetic engineering or gene editing. Finally, we focus on rice because of the global importance of this staple crop, especially in developing countries, and because the majority of rice breeding occurs in the public sector. Furthermore, rice is a model crop species, and therefore, research findings in rice can generally be transferred to other species.

### 2.3 Non-molecular breeding methods

Based on fundamental selection theory in plant breeding, shortening the length of time required for line development (i.e. the development of new breeding lines) regardless of the method used increases the rate of genetic gain. Importantly, quicker breeding and shorter breeding cycles can be one of the most simple and effective ways to develop new varieties that are adapted to current climates to minimise the effects of climate change [49].

#### 2.3.1 Rapid generation advance (RGA)

Based on a recent global survey, about 78% of rice breeders around the world claimed to use the pedigree breeding method [50]. While this is a proven breeding method in rice, the method relies on growing all plant populations in the field, following the normal growing seasons. Rapid generation advance (RGA), which is also referred to as single seed descent (SSD), is an alternative, faster breeding method that has been used by cereal crop breeders for decades. Essentially this method enables the line fixation to be performed more quickly by manipulating growth conditions of plants such that flowering and seed set is induced faster than under normal conditions in the field during a typical crop growing season. The bulk-population method is another effective and resource-saving breeding method; however it does not lead to time savings. The advantages of RGA compared to other breeding methods are speed, technical simplicity, requirement of less resources and low costs [51,52]. Due to the superiority of this method compared to the pedigree method based on empirical testing, it was implemented on a large scale in IRRI’s irrigated breeding program. This shortened the variety development time and breeding cycle by about 2 years.

#### 2.3.2 Doubled haploidy (DH)

Doubled haploid (DH) populations are produced by regenerating plants by the induction of chromosome doubling from pollen grains, which greatly shortens the line fixation stage because completely homozygous lines are produced immediately [53]. This is performed in tissue culture laboratories and is only possible in species that are amenable to tissue culture (e.g. cereal species including rice). This method has been used in rice breeding for decades [54] and like RGA, is a proven breeding method that has led to the release of many rice varieties. However due to biological factors, it has been more difficult to generate doubled haploid populations for *indica* subspecies compared to *japonica* subspecies [55].

#### 2.3.3 Shuttle breeding

The shuttle breeding concept was originally developed by the International Maize and Wheat Improvement Center’s (CIMMYT) wheat breeding progam, and was popularised by Nobel laureate Dr. Norman Borlaug. This system essentially enabled an extra generation to be advanced each year by using a different field location. The wheat shuttle breeding program at CIMMYT used two different field locations in Mexico which permitted off-season breeding activities. Another advantage of this system was improved selection, because the field locations contrasted for a broad range of different diseases and environmental conditions [56]. A shuttle breeding program was initiated in rice in 1982 [57]. However, over the years, many logistical problems were encountered regarding difficulties in moving seed across international borders, mainly due to intellectual property related issues and protection of national germplasm. Despite these constraints, the private sector (especially in temperate regions) uses off-season nurseries.

### 2.4 Molecular breeding methods

In the past 30 years, the use of DNA (or molecular markers) as tools for selection in plant breeding has led to significant improvements in efficiency and the subsequent release of new varieties [58,59]. Marker-based screening is often more efficient than using conventional methods leading to improved accuracy, cost or time savings, or permitting screening that is not possible using routine phenotyping methods [60]. A major advantage of using markers is that homozygosity can be traced (or detected) very efficiently. The implementation of molecular breeding has been widely adopted on an extremely large scale in private sector breeding programs, where reports have indicated increasing rates of genetic gain [61,62]. Marker-assisted selection (MAS) has now been routinely implemented in breeding programs of major crops leading to many reports of marker-assisted variety development [63,64].

#### 2.4.1 Marker-assisted backcrossing

Backcrossing is a plant breeding method commonly used to incorporate a target gene into a popular variety. In most cases, the parent used for backcrossing has a large number of desirable attributes but is deficient in only a few characteristics. The use of DNA markers in backcrossing greatly increases the efficiency of selection. Essentially, marker-assisted backcrossing (MABC) permits highly efficient detection of the target gene or quantitative trait locus (QTL) and enables the original critical characteristics of the recipient variety to be retained to essentially “upgrade” the original variety [58]. This approach may also be used to combine multiple genes or quantitative trait loci into a single recipient (often referred to as “marker-assisted pyramiding”). Both theoretical and empirical testing has indicated that using DNA markers reduces the time required for varietal development by several years. Enhancing stress tolerance in rice has been advocated as a priority for rice breeding [65] and this approach has been successfully applied for improving flood, salinity and drought tolerance in rice [63,66,67]. It should be noted that a validated gene(s)/QTL(s) is a prerequisite for this method to be applied.

#### 2.4.2 Genomic selection

Advances in rice genomics have led to the development of whole-genome based molecular breeding strategies. One specific method called genomic selection (GS) has recently emerged [68]. Genomic selection is a complementary method to MAS which is based on making genomic predictions from very large numbers of DNA markers rather than focusing on specific genes or quantitative trait loci [69,70]. Pilot studies in rice, wheat and maize during the last decade have provided encouraging results to shorten breeding cycles and variety development times. Genomic selection has enormous potential to be used for accurate selection of complex traits such as yield, and to shorten the breeding cycle to increase the rate of genetic gain [71]. However in practice there are significant economic and technical obstacles for actual implementation in public sector breeding programs in developing countries. Furthermore, the most cost effective and efficient way to implement genomic selection needs to be evaluated prior to implementation [71,72].

### 2.5 Conditions for successful breeding

The success of any plant breeding program crucially hinges on access to foresight information on markets, environment and climate. Therefore, breeding programs should invest in market research to develop dynamic product profiles that anticipate future trends in international and domestic markets for the commodity and its by-products, dietary patterns, urbanization, labour and land markets, structural transformation of economies, domestic, regional and international policies, abiotic and biotic stress incidence and severity, environmental conditions and climate [45].

From a crop R&D and breeding perspective, considerable further “pre-breeding” research (i.e. upstream of breeding programs such as gene discovery, trait research, applied molecular genetics) is needed to provide future breeders with new genes, germplasm and pilot and validate new tools to breed for improved varieties that are adapted to future climates.

## 3 Costs and benefits of accelerated breeding

RGA and MABC generally require significant initial investments in infrastructure and operational costs. To look at investments in breeding technology from an economic perspective, it is important to consider both costs and benefits. We make a further distinction between reversible and irreversible benefits and costs [73] (Table 1). Irreversibility means the streams of benefits or costs do not cease after the project is terminated. Compared to the traditional and still widespread [50] pedigree method, the (reversible) gains from acceleration of breeding are (i) an increased rate of genetic gain [51], (ii) more responsive release of varieties, and (iii) higher benefits when discounted over time [74].

**Table 1:** Costs and benefits of accelerated breeding.

	Reversible	Irreversible
Benefits	Increased rate of genetic gain	Avoid long-term detrimental impact of hunger on human development
	More responsive release of varieties	
	Earlier (discounted) benefits	
Costs	Operational costs^a^	Infrastructure investment

a Net operational costs may go up or down depending on the breeding technology.

### 3.1 Reversible costs and benefits

A large part of past improvements in yield can be attributed to successes in breeding modern rice and wheat varieties that were widely adopted during the “Green Revolution” [75]. Therefore, breeding is a logical solution to answering future food demand. Yet, plant breeding has often been referred to as an “endless task”, because it will always be necessary to replace existing varieties with new ones due to improved economically important traits (especially yield and other agronomic traits). A range of experiments in self-pollinated cereals has been conducted to estimate the genetic gain for yield indicating the average annual rate of yield increase from new varieties was approximately 1%. Since most studies project an increase in food demand of more than the average annual genetic gain of 1% observed in rice, wheat, barley and oats [[24], [25], [26], [27], [28],^32^,^33^], an acceleration of the rate of productivity increase may be needed. As stated above, this will be required from improved varieties (i.e. genetics) and other technologies or improved systems, especially improved agronomy. Reducing the time of the breeding cycle is generally considered to be one of the simplest ways to increase genetic gain in crop varieties [51].

Additionally, because climate change will likely increase weather variability, more adapted and resilient varieties as well as a more responsive (i.e. more timely) release of these varieties will be needed to ensure stability of the food supply [47,49]. More resilient varieties and more responsive dissemination means breeders can respond better to unpredictable changes in crop production environments, climate or markets.

A third argument looks at the economic impact of accelerating the breeding process. Since both breeding (costs) and adoption (benefits) of enhanced crop varieties span several years and generally do not overlap, time needs to be explicitly taken into account through the process of discounting. In brief, given a positive discount rate, acceleration of breeding yields positive incremental benefits by achieving benefits early on in the variety’s lifetime compared to the pedigree method, for different crops such as wheat [76,77] and rice [78,79].

These findings can be generalised through an exact multiplicator, which can be used to calculate incremental benefits from earlier variety release [74]. More specifically, relative incremental benefits equal ${\left(1+i\right)}^{r}-1$ where *r* is the reduction in the breeding process (in years) and *i* the discount rate used [74]. For a two-year time reduction at a 5% discount rate, benefits from accelerated breeding are approximately 10% higher than benefits from conventional breeding. For medium-scale breeding programs, a commercially successful variety developed through accelerated breeding can generate between 1 and 10 million US dollars in additional benefits. For large-scale breeding programs, such as in India and China, incremental benefits of shorter breeding cycles in rice breeding can add up to 10 billion US dollars over a period of 20–25 years [74].

To relate increased incremental benefits to food security, it is important to understand where those benefits originally came from. The majority of impact assessments calculate benefits as the sum of consumer and producer surplus from the technology-induced shift in supply curve [74,80]. Consumer surplus represents the welfare of consumers (in monetary terms) resulting from consumption at a given market price. As breeding is a cost-saving and yield-enhancing technology, it will generally increase production levels and reduce prices. Consumer surplus then captures the combined effect of food availability and access: consumers pay less and can afford to buy more food. Producer surplus measures the benefits a producer gets from participating in a market. As producers in the developing world are often net consumers, this increased income increases their access to food.

To ensure net benefits of public breeding (i.e. societal benefits minus operational costs at the institutional level) are positive, detailed cost information is needed. While the benefits listed in Table 1 are not technology-specific and may be achieved by different accelerated breeding methods, the cost structure is specific to the technology used. Two studies have calculated the net benefits (excluding capital investment cost) from reducing the breeding cycle through MAS at CIMMYT [81,82]. For a medium-sized breeding program (new variety planted on 10,000 ha), they found a $130,000 increase in (discounted) beneﬁts over the life of a commercially successful variety developed by the MAS breeding scheme compared to the conventional scheme.

Still, even when societal benefits far exceed operational costs, due to budget constraints, breeders might be more compelled to keep institutional costs small rather than maximising net benefits. A recent study found that for a medium-sized breeding program (output of 1000 breeding lines), there is a $500,000 decrease in (discounted) operational cost at IRRI from using RGA compared to conventional pedigree breeding operations (including capital investment cost) [51].

### 3.2 Irreversible costs and benefits

As mentioned in the previous paragraph, most impact assessments do not consider investment costs—especially infrastructure—explicitly. This is problematic as resource scarcity is an important constraint to developing novel breeding approaches, particularly in developing countries. The lack of a greenhouse has been identified as a major reason holding rice breeders back from adopting RGA as their main breeding method [50]. This is consistent with the observation that not all costs in an investment project are reversible. Given benefits at the beginning of the project are often uncertain—they might or might not exceed the costs incurred—investors prefer to invest in projects whose costs are reversible. In case the project turns out to be unprofitable, the assets can then be sold off again to minimise the losses. If investment costs are irreversible (such as a greenhouse or molecular laboratory facilities), an investor is inclined to wait a little longer to acquire more information about the benefits [73]. This additional information gives the investors the possibility not to engage in the project if unprofitable instead of cancelling it later and incurring a loss; however, benefits are smaller (foregone) since they are postponed by the waiting period. Thus, if breeders perceive current methods to accelerate breeding to be uncertain, postponing investment is reasonable given the associated investment costs are irreversible.

However, not only some of the costs associated with accelerated breeding are irreversible, some of the benefits are too. Classical welfare analysis based on consumer and producer surplus [83,84] typically does not capture irreversible benefits in terms of food security. Therefore, an alternative and more direct way to measure the impact of breeding on food security is to express the benefits as disability-adjusted life years (DALYs) [[85], [86], [87]]. While availability and access are drivers of food security, DALYs capture the outcome of food insecurity, i.e. the burden of disease that is caused by hidden and chronic hunger [6,88]. More specifically, DALYs capture "person-years lost in a population owing to disability and shortened life” [89] as a consequence of adverse health outcomes.

Recently, awareness is growing about the long-term consequences of malnutrition and poor health in general [6]. A growing number of studies report how deprived health translates into lower educational attainment, poorer health and lower social and economic status later in life [[90], [91], [92]]. The adverse impacts are not only strongest when poor health is experienced by children, but also transferred to children of the next generation leading to intergenerational transmission of poverty [93]. Moreover, the damage caused by malnutrition is irreversible [94,95]. Restated, even if we were to achieve global food security today, the adverse effects of past food insecurity would continue to affect current generations until far into the future. By accelerating breeding, improvements in food security are realised earlier meaning an entire cohort of people will escape lasting negative impacts such as stunting and mental impairment, improving their economic status.

It has been shown that irreversible benefits reduce irreversible costs by the order of one [73]. In other words, while irreversible costs justify postponing a project, irreversible benefits counteract this effect. Furthermore, if the net irreversibility effect is positive, there are no economic gains from waiting or maintaining “business-as-usual”. Given that investment in breeding technology is limited to international and national research centres, while the benefits in terms of food security accrue to millions of undernourished people worldwide, postponing technologies that can accelerate breeding makes no economic sense. It is, therefore, expected that the sizeable benefits—both reversible and irreversible—largely outweigh irreversible investment costs, which warrants immediate adoption of accelerated breeding practices. Since individual breeders may not always have the authority to decide on adoption of breeding methods, breeding managers, institutes and funding agencies should carefully assess the benefits and costs—both reversible and irreversible—of accelerated breeding.

## 4 Advocating the wider implementation of RGA

Considering the factors discussed above, we propose that RGA is the most appropriate breeding method that could be implemented by public sector breeding programs in developing countries in the short term. The effectiveness of this method has been proven for several crop species [52] and a recent review of the use of RGA is available [51]. Of the methods considered above, it is by far the most technically simple and requires the least complicated and expensive resources. It is also greatly superior in terms of cost-effectiveness compared to other methods [51].

When implementing new methods or technologies, feasibility is a critical factor. Considering public sector breeding programs, the following factors are relevant: cost, resources, and the level of technical complexity. Based on these factors, we believe RGA is the most appropriate method that could be realistically implemented in the majority of public programs in the short term. Evidence to support this was the quick adoption of RGA at the Bangladesh Rice Research Institute (BRRI) after initial demonstration and collaboration with IRRI [51]. For breeding programs with current access to doubled haploid labs, there would be advantages to continue or start using this breeding method. However if a breeding program is considering implementing doubled haploid methods from the beginning, there are critical considerations such as prohibitive costs, requirement of expertise in tissue culture, specialist tissue culture laboratories and growth chambers [46]. The cost for RGA generated breeding lines is estimated to be significantly lower compared to doubled haploid (IRRI unpublished data). Furthermore, there are some cases where doubled haploid technology was adopted in wheat and barley breeding programs, but later discontinued because it was not economically viable [96]. A careful cost-benefit analysis should be conducted before implementation of doubled haploid technology [97].

In proposing RGA as the main breeding method, we must also consider factors that could impede adoption. Firstly, facilities such as screenhouses or greenhouses are usually required to protect breeding material from weather, pests and animals, and to be able to conveniently manipulate growing conditions. The initial funds to establish such facilities can be an obstacle in practice, although cost analysis indicated that the cost-savings to breeding programs may be quickly recovered (i.e. within a few years) after the initial investment [51]. It is also noteworthy that temperature control can be a major issue in greenhouses and pests and diseases can be more prevalent in screenhouse/greenhouse facilities so appropriate measures need to be taken.

In cases where resources are scarce, it could be possible to implement RGA on a limited scale as a secondary breeding method. In our view, this configuration would still enable achieving the objectives of accelerated breeding (for example, a breeding “express lane” could be established for the highest priority populations). In other cases, RGA can be conducted directly in the field (referred to as “Field RGA” [51]), when the annual temperatures permit continuous planting (e.g. tropics or sub-tropics).

A recent global survey of plant breeders revealed that most rice breeders were aware of the theory and advantages of RGA and there was a high willingness to adopt [50]. However, this did not translate into actual adoption. This implied that further published empirical evidence and training or extension-related activities such as workshops could be required to influence attitudes and adoption as credibility and awareness towards RGA were important determinants of adoption behaviour.

Finally, our advocacy is based on the assumption that RGA would be integrated into “best practice” or effective breeding programs. For example, for RGA to be effective, there need to be several efficient components in place such as genetic variation for key target traits, reliable screening methods, and a multi-location trial testing system. The RGA breeding method would not deliver if it was simply incorporated into an ineffective breeding program.

### 4.1 RGA and beyond

Higher levels of sophistication are possible to further accelerate generation advancement for crops. Recently, there has been considerable interest in “speed breeding” across a wide range of crop species to achieve greatly accelerated generation times by manipulating day-length with artificial lighting in fully enclosed, controlled environment growth chambers [[98], [99], [100]]. However due to the facilities required and higher running costs, it is unlikely that these systems could be implemented in the public sector in developing countries on a large scale in the short term, although these methods have considerable potential to be applied for “fast-tracking” new varieties if there was access to such facilities or service provider.

An improved breeding method would be to use MAS during RGA as this would permit accurate selection for traits with well-characterised genes. We previously proposed that molecular breeding can be conveniently incorporated into RGA systems. An ideal situation would be to integrate faster breeding methods with MAS and genomic selection, to obtain synergies from the combined use of these methods [48,49,71,100]. Such new breeding schemes (i.e. combinations of multiple technologies) and many parallel research and development activities would be required if worse than expected effects from climate change are encountered.

## 5 Conclusion

Population growth, climate change and economic growth are posing serious challenges to food availability rendering current food production practices insufficient to meet future food demand. Plant breeding, which has played an important role in alleviating hunger in the past, is typically characterized by long production processes facing rapidly changing market, environmental and climate conditions. We argue that acceleration of the breeding process is needed to make plant breeding more responsive to constantly moving targets and sustain its role as principal provider of food security.

We propose RGA as the most appropriate breeding method that could be implemented by public sector breeding programs in developing countries in the short term. There is considerable empirical evidence proving the effectiveness of this method. RGA is also by far the most technically simple accelerated breeding method, it requires relatively little financial and human capital to operate and it is greatly superior in terms of cost-effectiveness compared to other methods. Breeders also have a relatively high level of awareness about RGA and its benefits although further published empirical evidence and training or extension-related activities are needed.

Because of the existence of both reversible and irreversible benefits and costs, additional measures (such as DALYs) are required to assess the impact of accelerated plant breeding on food security. Empirical evidence on RGA suggests operational benefits for rice breeding exceed investment costs after already a few years. A lack of investment in accelerated breeding will not only lead to considerable forgone benefits from earlier adoption, it will also bear societal costs due to the long-term detrimental impact of hunger on human development. In sum, the large irreversible and reversible benefits indicate that postponing technologies that can accelerate plant breeding makes no economic sense and warrant immediate adoption of accelerated breeding practices. Breeding managers and research directors should facilitate the implementation of such technologies.

This century, the challenges for breeders and crop scientists could be unprecedented, and so it is hoped that as has happened in the past, technological innovation will ensure sufficient food production for the global population. Technology adoption can take years to implement in practice. It is hoped that the justification discussed in this paper will encourage public sector breeders to implement faster breeding methods in the short term in order to mitigate risks from future scenarios.

However, even with the release of the newest improved varieties, there will always be a need for strong agronomy research and extension and strong seed systems. Plant breeding is only one—albeit important—element in the larger food production system. Alternative approaches to increase food production as well as policies to ensure sustainability of food production, including mitigation strategies, need to be considered at the same time. Foresight information on markets, environment and climate, pre-breeding research and particularly successful dissemination strategies are needed as well since low levels of adoption severely limit improved varieties’ potential to improve food security.
